# Systolic Dyssynchrony Index: A Possible Robust Parameter for Risk Stratification of SCD in HCM?

**DOI:** 10.1016/j.jacasi.2025.06.002

**Published:** 2025-07-22

**Authors:** Yuri Ochi, Toru Kubo, Hiroaki Kitaoka

**Affiliations:** Department of Cardiology and Geriatrics, Kochi Medical School, Kochi University, Kochi, Japan

**Keywords:** hypertrophic cardiomyopathy, mechanical dyssynchrony, risk assessment, ventricular tachycardia

**Figure:**
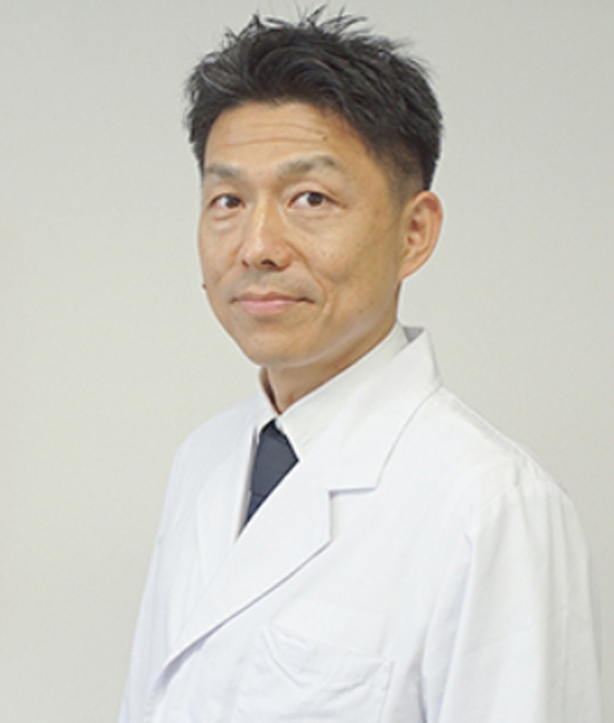

Hypertrophic cardiomyopathy (HCM) is characterized by genetic heterogeneity, diverse clinical phenotypes, and a highly variable natural history. Sudden cardiac death (SCD), the most devastating and feared complication of HCM, is caused mainly by malignant ventricular tachycardias (VTs). Several guidelines have recommended that all patients with HCM should undergo risk assessment for SCD to identify those able to benefit from implantable cardioverter-defibrillator (ICD) implantation.^1^, ^2^, ^3^, ^4^ The European Society of Cardiology (ESC) and the American Heart Association/American College of Cardiology have each published major guidelines for ICD implantation in patients with HCM.^2^, ^3^, ^4^ These guidelines are in agreement regarding the Class 1 indications of ICD implantation for secondary prevention. However, the indications of ICD implantation for primary prevention are controversial. It has been reported that even when the traditional risk factors of SCD raised in the guidelines are absent, some patients with HCM present with documented ventricular arrythmias and sudden cardiac collapse.^5^ Because it remains challenging to determine an individual’s risk of SCD, additional risk markers have been investigated with the aim of improving current risk stratification strategies.

In this issue of *JACC: Asia*, Wang et al^6^ reported that systolic dyssynchrony index (SDI) measured with 3-dimensional echocardiography (3DE) showed potential as a new predictive indicator for nonsustained ventricular tachycardia (NSVT) and SCD in a large-scale cohort of Chinese patients with HCM. They prospectively measured comprehensive echocardiographic parameters of mechanical dyssynchrony and strain values of the left ventricle (LV) in the 3 main spatial directions through 2-dimensional echocardiography (2DE) and 3DE in 610 patients with HCM (median age 51.0 years, 33.3% were female). Mechanical dyssynchrony was assessed by mechanical dispersion (MD) with 2D speckle-tracking echocardiography (STE), and SDI was evaluated by 3DE. Subsequently, patients underwent 24-hour Holter monitoring to detect NSVT, and cardiac magnetic resonance (CMR) to evaluate the extent of late gadolinium enhancement (LGE). Wang et al^6^ also investigated the capability of SDI to predict NSVT for each of the ICD recommendation classes advocated by the 2022 ESC guideline,^2^ 2023 ESC guideline,^3^ and 2024 American College of Cardiology/American Heart Association guideline.^4^

The major findings of that study were as follows: 1) patients with HCM exhibited mechanical dyssynchrony of the LV, which was more pronounced in the presence of NSVT; 2) SDI value had greater capability than MD for the detection of NSVT and showed an independent association with occurrence of NSVT; and 3) SDI could be used to distinguish Class 2, Level of Evidence: A from the Class 2, Level of Evidence: B and Class 3 ICD implantation recommendations of the 2022 and 2023 ESC guidelines.

In patients with HCM, myocardial fibrosis and disarray induce heterogeneity of ventricular activation and ventricular contraction. These anatomical and electrical substrates cause mechanical dispersion and generate ventricular arrhythmias.^7^ Although it is not possible to evaluate the underlying anatomical and electrical substrates of ventricular arrhythmias directly, several studies have shown that patients with more significant myocardial fibrosis detected by LGE-CMR have an increased risk of NSVT and SCD.^7^^,^^8^ Regarding echocardiographic parameters in patients with HCM, global longitudinal strain (GLS) and MD evaluated by 2D-STE are associated with NSVT and LGE-CMR.^7^, ^8^, ^9^, ^10^ The new finding in the study by Wang et al^6^ is that SDI measured by 3DE was more closely related to NSVT compared to MD, GLS-2D, GLS-3D, global radial strain–3D, and global circumferential strain–3D. Wang et al^6^also showed a relationship between high SDI values and the presence or the percentage of LGE by CMR. In general, assessment of NSVT by Holter monitoring carries the risk of missing and underestimating events inherent to the short examination time, and the assessment of LGE by CMR can be difficult to apply in daily practice due to its limited availability or the patient’s renal dysfunction. SDI by echocardiography may help to fill these clinical gaps. In addition, a key strength of the study is the detailed assessment of the relationship between SDI and the ICD recommendations of the current major guidelines. A high value of SDI could potentially enable identification of patients who fall into the Class 2, Level of Evidence: A ICD recommendation.

However, we have some concerns regarding whether SDI can be applied in a clinical setting. SDI has been already reported for over a decade as a marker of LV global dyssynchrony for cardiac resynchronization therapy,^11^^,^^12^ but SDI is not yet in widespread use in clinical practice. In addition, the strain value is influenced by factors such as age, body weight, and LV length and volume and is highly dependent on the platform and software used. Compared with 2D-STE, 3DE can more precisely and accurately reflect local changes in the heart, and single-cardiac-cycle 3DE can solve the problem of splicing in multicardiac-cycle 2DE.^13^ Nevertheless, there are limited data for the normal range of SDI in the overall population, and the cutoff value of SDI varies widely among reports.^11^^,^^12^ Variable wall thickness in patients with HCM makes fitting the region of interest in STE particularly challenging. Larger, prospective studies are required to establish the standard specification of SDI before it can be considered as an additional marker for SCD risk assessment for HCM.

In summary, the findings of the study by Wang et al^6^ have enhanced the potential of SDI for evaluation of SCD risk in patients with HCM. We expect that the use of SDI in addition to standard risk markers will improve the power to predict SCD in these patients.

## Funding Support and Author Disclosures

The authors have reported that they have no relationships relevant to the contents of this paper to disclose.
